# Dynamic Light Scattering Study of Inhibition of Nucleation and Growth of Hydroxyapatite Crystals by Osteopontin

**DOI:** 10.1371/journal.pone.0056764

**Published:** 2013-02-14

**Authors:** John R. de Bruyn, Maria Goiko, Maryam Mozaffari, Daniel Bator, Ron L. Dauphinee, Yinyin Liao, Roberta L. Flemming, Michael S. Bramble, Graeme K. Hunter, Harvey A. Goldberg

**Affiliations:** 1 Department of Physics and Astronomy, University of Western Ontario, London, Ontario, Canada; 2 Department of Chemistry, University of Western Ontario, London, Ontario, Canada; 3 School of Dentistry and Department of Biochemistry, University of Western Ontario, London, Ontario, Canada; 4 Department of Earth Sciences, University of Western Ontario, London, Ontario, Canada; University of Notre Dame, United States of America

## Abstract

We study the effect of isoforms of osteopontin (OPN) on the nucleation and growth of crystals from a supersaturated solution of calcium and phosphate ions. Dynamic light scattering is used to monitor the size of the precipitating particles and to provide information about their concentration. At the ion concentrations studied, immediate precipitation was observed in control experiments with no osteopontin in the solution, and the size of the precipitating particles increased steadily with time. The precipitate was identified as hydroxyapatite by X-ray diffraction. Addition of native osteopontin (nOPN) extracted from rat bone caused a delay in the onset of precipitation and reduced the number of particles that formed, but the few particles that did form grew to a larger size than in the absence of the protein. Recombinant osteopontin (rOPN), which lacks phosphorylation, caused no delay in initial calcium phosphate precipitation but severely slowed crystal growth, suggesting that rOPN inhibits growth but not nucleation. rOPN treated with protein kinase CK2 to phosphorylate the molecule (p-rOPN) produced an effect similar to that of nOPN, but at higher protein concentrations and to a lesser extent. These results suggest that phosphorylations are critical to OPN’s ability to inhibit nucleation, whereas the growth of the hydroxyapatite crystals is effectively controlled by the highly acidic OPN polypeptide. This work also demonstrates that dynamic light scattering can be a powerful tool for delineating the mechanism of protein modulation of mineral formation.

## Introduction

Biomineralization is the process by which living organisms produce the minerals that make up hard tissues such as shell, bone, and teeth [1]. The process of mineral formation *in vivo* is complex and involves several stages [2]. The details remain incompletely understood, but certain proteins are believed to play central roles both in the initial nucleation of biomineral crystals and in the regulation of their subsequent growth, with some proteins acting to inhibit nucleation and growth and others to enhance it [2]–[4]. While controlled biomineralization is essential to human life, pathological biomineralization can lead to serious medical conditions such as kidney stones, atherosclerosis, and joint arthropathy [5]. There is thus substantial motivation for the development of a more complete understanding of the biomineralization process and of the regulatory roles played by specific proteins.

Hydroxyapatite, (HA, Ca_10_(PO_4_)_6_(OH)_2_), is the principal mineral component of bone and teeth, making up approximately 65% by weight of bone and dentin and 95% by weight of dental enamel [6]. The extracellular fluid in vertebrates is supersaturated with respect to hydroxyapatite and other calcium phosphates, but spontaneous precipitation is inhibited by regulatory proteins. It has been proposed that the solid phase initially precipitates as small amorphous clusters of calcium and phosphate ions, which transform eventually to crystalline HA as they grow [6]. Biomineralized HA can form both plate-like and rod-like crystals, depending on the environment, with the crystal structure again controlled by proteins [3]. Although natural bone mineral includes a significant fraction of ionic substitutions and the corresponding structural imperfections, HA itself is typically used as a model for biological mineralization.

Osteopontin (OPN) is an acidic glycoprotein that has been shown to act as an inhibitor of HA crystal growth and is believed to play a role in preventing soft-tissue calcification [7], [8], [9]. It is a flexible, disordered protein approximately 300 amino acids in length [10]. Because it contains a high percentage of aspartic acid and glutamic acid (including a contiguous aspartic acid sequence), OPN is strongly negatively charged under physiological conditions. Post-translational modifications further increase the negative charge density. Recombinant rat OPN has a pI of 4.5, while native OPN from rat bone, which has an average of 10 phosphate groups per molecule [11], [12], has a pI of 4 [13]. OPN is found in a variety of tissues, including bone, kidney, brain, and smooth muscle, as well as various biological fluids [5]. It is also abundant in pathological calcifications such as atherosclerotic plaque and kidney stones [5].

*In vitro* studies have shown OPN or synthetic peptides based on OPN sequences to be potent inhibitors of both the nucleation [8], [9], [14] and growth [15] of HA crystals. These molecules have also been shown to inhibit the formation of other biominerals including calcium carbonate [16] and both the mono and dihydrate forms of calcium oxalate [7], [17]. The mechanism by which OPN inhibits mineral formation and growth is still a subject of debate and significant ongoing research. Modification of aspartic and glutamic acid reduces the HA-inhibiting potency of OPN [9]. The phosphorylations have been shown to play an important role in OPN's inhibitory activity, with non-phosphorylated OPN or OPN peptides being much less inhibitory than the native protein or peptide [9], [18], [19]. In general, however, it appears that inhibitory potency correlates with overall acidity rather than particular chemical groups [14], [15].

Most previous laboratory experiments on the inhibitory potency of proteins have used either autotitration or double-diffusion gel methods. The former method uses a feedback system to inject titrant into a calcium phosphate solution, maintaining the *p*H constant as crystals grow in the solution [9], [20]. A modification of this protocol is the constant-composition assay, which maintains calcium and phosphate concentrations fixed in addition to *p*H [14], [15], [21]. In both systems inhibitor proteins decrease the rate of crystal growth, resulting in a lower rate of titrant injection. These methods have been successfully used to assess the inhibitory potency of OPN and other bone-related proteins [20], [22]. These experiments are technically challenging and time-consuming, however, with individual runs taking several hours.

In double-diffusion methods, solutions containing calcium and phosphate ions are continuously pumped past opposite ends of agarose, collagen, or gelatin gels. Ca^2+^ and PO_4_^3−^ ions diffuse into the gel, and nucleation and crystal growth occur within the gel where the ion concentrations are appropriate. Incorporating a protein into the gel allows its effect on the mineralization process to be studied. This method has been used to assess the nucleation potency of several bone-related proteins [20], [23], but it is even more time-consuming, with runs taking days, and provides little or no information about the time-dependence of the process.

Previous studies have shown the utility of dynamic light scattering (DLS) for investigating the nucleation and growth of nanometer-sized particles, including minerals. The technique is relatively fast, particularly in comparison with the traditional methods described above, and provides quantitative assessments that cannot be readily obtained using those techniques. In particular, DLS allows us to monitor the early stages of the precipitation process and determine the size and relative concentration of the growing crystals as a function of time, thus giving crystal growth rates directly and allowing an immediate assessment of the inhibitory effects of the proteins.

Onuma *et al.* [24] used a continuous-angle laser light-scattering instrument to measure precipitation kinetics of HA from supersaturated solutions. Earlier studies by the same group used DLS to demonstrate the presence of nanometer-sized calcium phosphate clusters that remained stable over time [25], [26]. The addition of fetuin A to a solution of calcium phosphate caused changes in the dynamics of the precipitation that were studied with DLS [27]. Calcium carbonate precipitation has also been monitored by DLS; addition of lanthanum ion caused inhibition of growth and a reduction in the size of the particles [28].

In this paper we use DLS to study the kinetics of the nucleation and growth of calcium phosphate crystals in the presence of native rat-bone OPN, recombinant OPN (which lacks the post-translational phosphorylations of the native protein), and phosphorylated recombinant OPN. We perform complementary measurements on the native and recombinant proteins using a constant-composition autotitration protocol, and compare the results obtained with the two techniques.

## Methods

### Protein Preparation

Native OPN (nOPN) was extracted from rat bone and purified using protocols adapted from Zhang *et al.* [29] All procedures were approved by the Council on Animal Care of the University of Western Ontario and were in accordance with the guidelines of the Canadian Council on Animal Care. The rat nOPN, with an approximate mass of 37,600 g/mol based on matrix-assisted laser desorption time-of-flight mass spectrometry (MALDI-TOF MS) contains an average of 10 phosphate residues per molecule and is also glycosylated and sulfated [12]. Recombinant rat osteopontin (rOPN) with a mass of 36,046 g/mol, consisting of the polypeptide chain without post-translational modifications but containing a His-tag and linker sequence, was expressed in *E. coli* and purified as previously described [13]. Phosphorylated rOPN (p-rOPN) was prepared by treating rOPN with the protein kinase CK2 following a protocol similar to that described in Ref. [30]. This resulted in a broad series of peaks on MALDI-TOF MS ranging from 36,287–37,088 g/mol, equivalent to 3–13 phosphate groups per OPN molecule, with an average of 7–8. Protein content was determined by amino acid analysis with inclusion of carboxymethylcysteine as internal control for recovery, undertaken at the Advanced Protein Technology Centre, Amino Acid Analysis Facility, at The Hospital for Sick Children, Toronto, Canada. The proteins were stored as pre-weighed, freeze-dried aliquots, and were freshly dissolved into a volume of filtered 0.05 M Tris-HCl buffer solution at *p*H 7.4 as required.

### Solution preparation

All chemicals were purchased from Sigma Aldrich and had purities of at least 99%. Concentrated stock solutions of 0.25 M sodium phosphate dibasic (Na_2_HPO_4_); 2 M sodium chloride (NaCl); 1 M Tris-HCl; and 0.5 M calcium chloride (CaCl_2_) were prepared using distilled deionized water. All solutions were filtered through 0.2-µm membrane filters. The stock solutions were stored in clean, autoclaved glass bottles.

Three working solutions were prepared daily from the stock solutions: 12 mM CaCl_2_, 150 mM NaCl, 18.75 mM Tris (Ca solution); 7.5 mM Na_2_HPO_4_, 150 mM NaCl, 18.75 mM Tris (phosphate solution); and 18.75 mM Tris, 150 mM NaCl (Tris solution). After preparation, the *p*H of each solution was adjusted to 7.40 using dilute NaOH or HCl and the volume adjusted to 100 ml. The solutions were then filtered again through a 0.02-µm membrane filter to remove dust or particulate contaminants that could scatter light. Working solutions were used within 24 h of preparation.

For each run, the desired concentration of the test protein was dissolved in 100 µl of Tris solution. This was then combined with 850 µl of each of the Ca^2+^ and phosphate solutions in a 5-ml borosilicate glass tube, giving a total volume of 1.8 ml and Ca^2+^ and phosphate concentrations of 5.67 mM and 3.54 mM, respectively. These concentrations were chosen because they resulted in precipitation of crystals with a growth rate that was slow enough to permit accurate measurements, but fast enough that significant growth occurred over the forty-minute duration of the experiments. The tube was gently shaken to mix the solution, then quickly inserted into the sample chamber of the light scattering instrument. In the absence of protein, precipitation started immediately once the Ca and phosphate solutions were mixed together.

We found that our results varied slightly from day to day. To minimize this, all data for a given protein were taken over a period of five to six hours on the same day using the same buffers and the results compared with control runs taken on the same day.

### Identification of the Precipitate

The material that precipitated from our solutions was extracted and identified using X-ray diffraction. Since the amount of precipitate produced from a single 1.8 ml sample (as described above) was very small, we prepared several replicate samples in parallel. After the solutions had been allowed to incubate for 40 minutes (the duration of our light scattering experiments), their contents were transferred to microfuge tubes and centrifuged at 8000 *g* for 2 min. The supernatant fluid was removed and the remaining precipitate washed with 100 µl of *p*H-10 water, then centrifuged for an additional 2 min. The supernatant was again removed and 100 µl ethanol added to the tubes. After gentle shaking to wash the crystals, the contents of all microfuge tubes were combined into a single tube and spun for 2 min. The ethanol was removed and the collected precipitate dried at room temperature, then placed in a dessicator. Roughly 1 mg of the precipitate was analyzed with a Bruker D8 Discover micro X-ray diffractometer using Cobalt Kα radiation (1.78897 Å) and identified by comparison of the measured diffraction pattern with catalogued standard patterns from the International Centre for Diffraction Data (ICDD) database. Details of the X-ray diffraction technique are provided in [31].

### Dynamic Light Scattering

Dynamic light scattering is a well-established technique for particle sizing, and the theoretical background is laid out in detail in a number of texts [32], [33]. Our experiments were performed using an ALV CGS-3 goniometer-based light scattering system [34]. The sample under study is contained in a cylindrical glass tube positioned on the axis of a goniometer system. Polarized light from a 20-mW HeNe laser with a wavelength λ = 632.8 nm is directed through the sample, and a photomultiplier measures the intensity *I* of the scattered light at an adjustable angle θ from the direction of the incident beam. The experiments reported here were all carried out at θ = 90°. The sample cuvette is surrounded by a vat containing toluene, which has a refractive index close to that of glass and so minimizes stray scattering from the wall of the cuvette. The particles undergo Brownian motion within the suspending fluid, giving rise to temporal fluctuations in *I*. The autocorrelation function of the scattered intensity is 
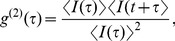
(1)


where the angle brackets indicate a time average and τ is the lag time. Assuming that the scattering is a random process, 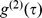 is related to the autocorrelation function of the scattered electric field 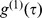 by the Siegert relation [32],
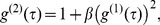
(2)


where *β* is a constant that depends on the size of the detector and the details of the experimental optics. Due to the random motion of the scattering particles, 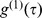 will decay as τ increases, and the hydrodynamic radius of the scatterers can be determined from the decay rate.

For monodisperse spherical scatterers, the decay of 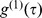 is exponential. The autocorrelation functions measured in our experiments did not show a simple exponential decay, however, as discussed below. We determined 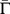, the mean relaxation rate of 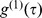, and its second moment 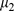 by fitting the measured intensity autocorrelation function to the first terms of a cumulant expansion of the form [35]

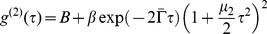
(3)


using a standard nonlinear least-squares fitting routine. This form assumes that the scatterers have a Gaussian distribution of sizes with a mean hydrodynamic radius of 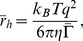
(4)


where 
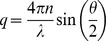
 is the magnitude of the scattering wave vector, *n* the refractive index of the scattering medium, and *θ* the scattering angle, and a standard deviation of



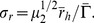
(5)


The hydrodynamic radius 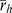 is the radius of a sphere that would have the same average diffusion constant as the scattering particles. The particles which precipitate from solution in our experiments are almost certainly not spherical, in which case the values of 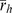 determined from light scattering measurements can be taken as a characteristic size.

Our light scattering measurements were carried out over a period of 40 minutes after each sample was prepared. The intensity autocorrelation function 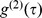 and the mean scattered intensity 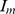 were determined from measurements accumulated over 30 second intervals. All measurements were carried out at room temperature.

### Constant Composition/Seeded-Growth Analysis

The constant-composition protocol developed by Tomson and Nancollas [21] was used with modifications as described in [15]. Briefly, HA seed crystals are incubated with various concentrations of protein in a solution containing 0.5 mM Ca(NO_3_)_2_, 0.3 mM Na_2_HPO_4_, 150 mM NaCl, and 0.25 mg/ml HA. The HA seed crystals were prepared as in Ref. [36] and shown by the Brunauer-Emmett-Teller method to have a surface area of 84.1 ± 0.1 m^2^/g. The seed-crystal suspension was maintained at 37 °C, with contents kept under a nitrogen atmosphere throughout the incubation. The pH was adjusted to 7.40 ± 0.01 prior to the start of the incubation, and maintained at that value by the simultaneous addition of solutions containing 1.6 mM NaOH and 2.1 mM Na_2_HPO_4_ from one buret and 3.5 mM Ca(NO_3_)_ 2_ and 300 mM NaCl from a second buret. The volume of added titrant, which is a measure of the growth of the HA crystals, was monitored over the 4 h incubation period and used to determine the crystal growth rate.

## Results

### Dynamic Light Scattering

#### A: Control experiments

**Figure 1:**
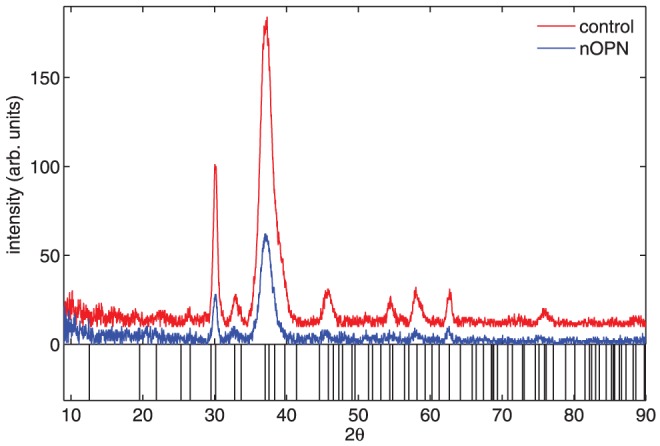
X-ray diffraction pattern of precipitate recovered from the solutions as described in the text. Red: control experiments, no protein; blue: [nOPN]  =  1 µg/ml. The control data have been offset vertically for clarity. The positions of the peaks in 2*θ* using Cobalt Kα radiation in the reference pattern for hydroxyapatite are shown at the bottom of the figure (ICDD card #01-073-0293).

Control experiments using the solutions described above, but with no protein added, were performed regularly to provide baseline data and to confirm consistency of the results. X-ray diffraction measurements on the precipitate that formed in control experiments produced a diffraction pattern that matched that of hydroxyapatite. Textural observations from the measured two-dimensional diffraction pattern show the crystals to be fine-grained (homogeneous Debye rings) but well-crystallized (narrow diffraction lines). The two-dimensional data were integrated to produce a conventional one-dimensional pattern, as shown in Figure 1.

**Figure 2:**
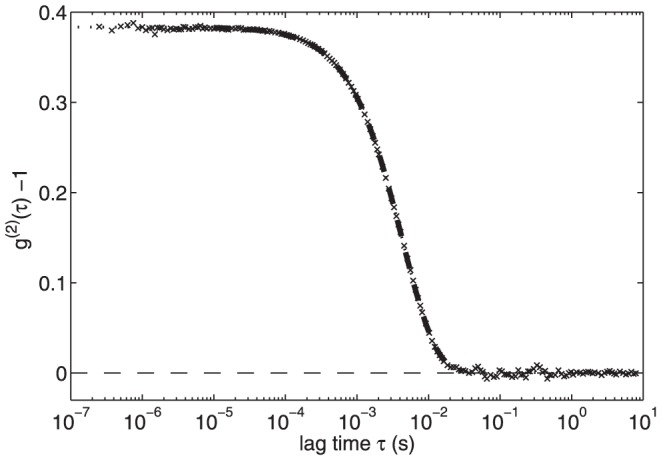
A typical intensity autocorrelation function, 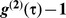, obtained from a control experiment with no protein. The heavy dashed curve is a fit to the cumulant expansion given in Eq. (3), performed on data with 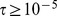 s and 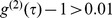 to minimize the influence of noise at short and long lag times. The dotted curve is an extrapolation of the fit over the rest of the measured *τ* range.

Figure 2 shows a typical intensity autocorrelation function obtained from a DLS experiment with the control solution. A fit to Eq. (3) is shown in Figure 2 and describes the data well. For these particular data, recorded 23 minutes into the control run, the fit gives a mean hydrodynamic radius of 630 nm and a standard deviation of the distribution of particle sizes of 240 nm. Both the high quality of the fit seen in the figure and the implied broad distribution of particle sizes are quite typical. As a check, we also analyzed the decay of 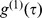 using a regularized inverse Laplace transform procedure (the so-called CONTIN analysis) [37], [38]. This method gave a distribution of particle sizes with a single broad peak, fully consistent with the results obtained from the cumulant fits. We used the cumulant method to analyze the data presented below.

**Figure 3:**
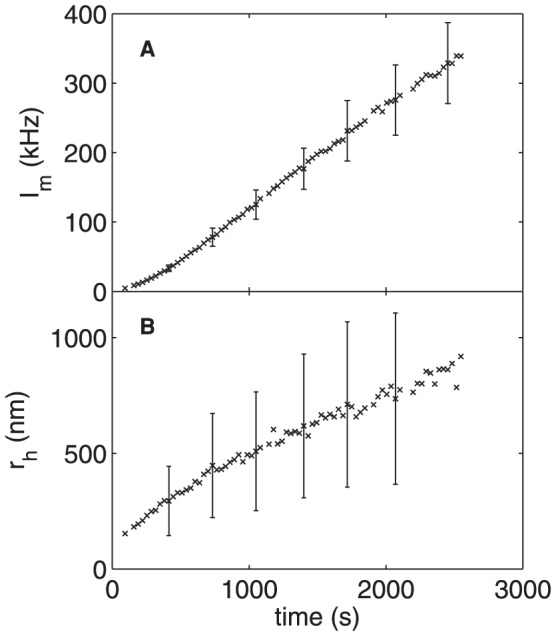
Precipitation of calcium phosphate in the absence of protein. (A) Scattered photon count rate 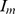 and (b) hydrodynamic radius 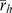 obtained from an experiment with no added protein. The error bars in (A) indicate typical values of the standard deviation of the count rate. In (B) they indicate the standard deviation of the distribution of particle sizes determined from fits of Eq. (3) to the intensity autocorrelation function. Both the count rate and 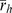 increase over the course of the experiment as the particles of calcium phosphate precipitate grow.

Results from a typical control experiment are shown in Figure 3. Figure 3A shows the mean scattered light intensity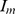, given in terms of the photon count rate detected by the photomultiplier, as a function of time *t*. No useful data could be obtained during roughly the first two minutes of the run due to the fluid motion induced when the sample was mixed and placed in the goniometer. The scattered intensity, which depends on both the size and the concentration of the scattering particles [32], increases steadily and roughly linearly over the course of the run. The particle size can be determined from the decay of the autocorrelation function, as described above. Figure 3B shows the mean hydrodynamic radius of the scatterers and the standard deviation of the size distribution for the same control run. 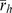 is approximately 150 nm at *t* = 94 s, when the first data were obtained, and increases to about 900 nm over the run. In this run, the growth rate of 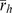 – *i.e.*, the slope of the plot in Figure 3B – decreases from about 0.4 nm/s at early times to 0.17 nm/s at the end of the run.

#### B: nOPN

**Figure 4:**
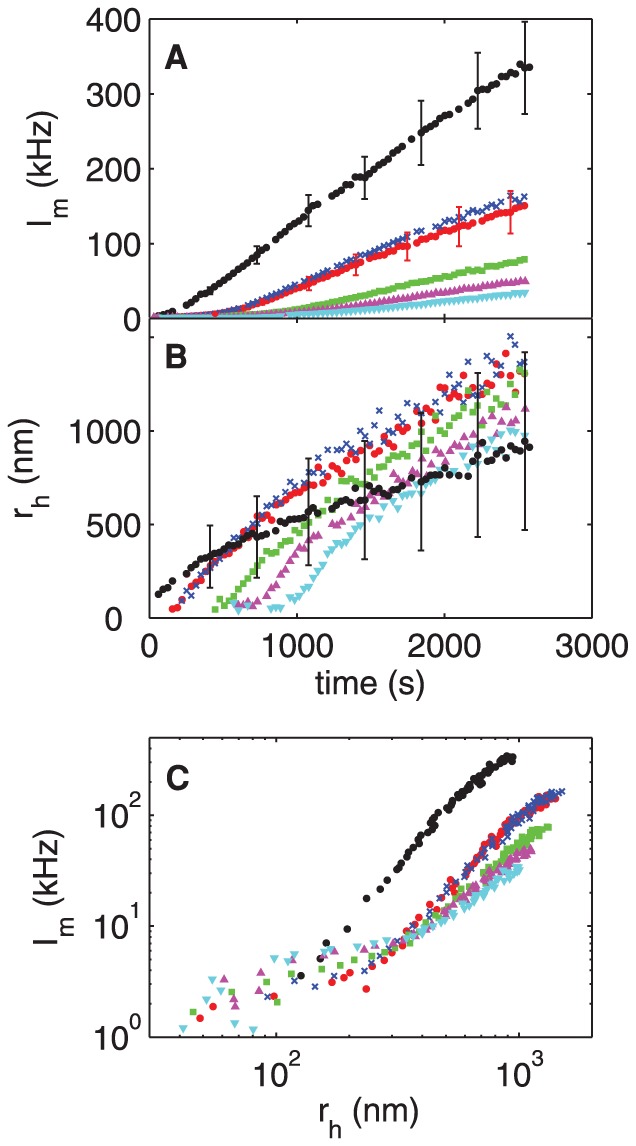
Precipitation of calcium phosphate in the presence of nOPN. (A) Mean scattered intensity and (B) hydrodynamic radius plotted as functions of time for a range of concentrations of native osteopontin, nOPN. (C) shows 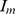 as a function of 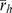. black circles: control, [nOPN]  =  0 µg/ml; red circles: 1 µg/ml (0.03 µM); crosses: 1.4 µg/ml (0.05 µM); squares: 2 µg/ml (0.06 µM); upward-pointing triangles: 3 µg/ml (0.09 µM); downward-pointing triangles: 4 µg/ml (0.12 µM). Error bars in (A) indicate typical standard deviations in the photon count rate for the control run and for [nOPN]  =  1.0 µg/ml. In (B), they show the standard deviation 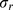 of the distribution of particle sizes for the control run. The values of 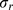
in the protein runs are similar.

Figure 4 shows the scattered intensity and the hydrodynamic radius measured in experiments with a range of concentrations of nOPN added to the calcium phosphate solution. The presence of nOPN at a concentration as low as 1 µg/ml (∼ 0.03 µM) significantly decreases the scattered light intensity, as illustrated in Figure 4A, and delays the growth of the particles. Both the scattered intensity and 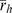 (shown in Fig. 4B) remain very low for a period of time that increases as the concentration of nOPN increases, but eventually both start to increase. Once the particles start to grow, however, 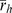 increases more quickly in the presence of nOPN than in the control experiments, and after a sufficient time the mean size of the precipitating particles in fact becomes larger than at the same time in the control experiments. Despite this, the scattered intensity remains significantly below that of the control experiment for the full duration of our measurements. The scattering cross-section is much higher for larger particles than for smaller particles. The fact that 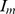 remains much lower than the control value therefore indicates that the concentration of scatterers is much smaller in the presence of nOPN. This is confirmed by the fact that much less precipitate was recovered per 1.8 ml tube of solution in the nOPN experiments than in the control experiments. The crystals precipitated in the presence of nOPN show a similar X-ray diffraction pattern to those formed in the control experiments, as illustrated in Figure 1, and are again identified as HA. These results imply that nOPN dramatically inhibits the nucleation of CaPO_4_ clusters. The few particles that do nucleate, however, grow more quickly in the presence of nOPN than in the control experiments.

The effect of the protein on the concentration of precipitating particles is illustrated more clearly in Figure 4C, in which the scattered light intensity is plotted as a function of 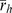. If we assume that the distribution of particle sizes at a given value of 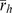 is independent of the protein concentration, then at a fixed value of 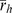, the intensity 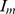 will depend only on the concentration of scatterers. Figure 4C thus shows that the number density of scattering particles with 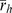 larger than about 200 nm is roughly a factor of five smaller in the presence of nOPN than in the control experiment. Interestingly, when nOPN is present, the concentration of particles decreases only slightly with increasing [nOPN] over the range of protein concentrations studied here.

**Figure 5:**
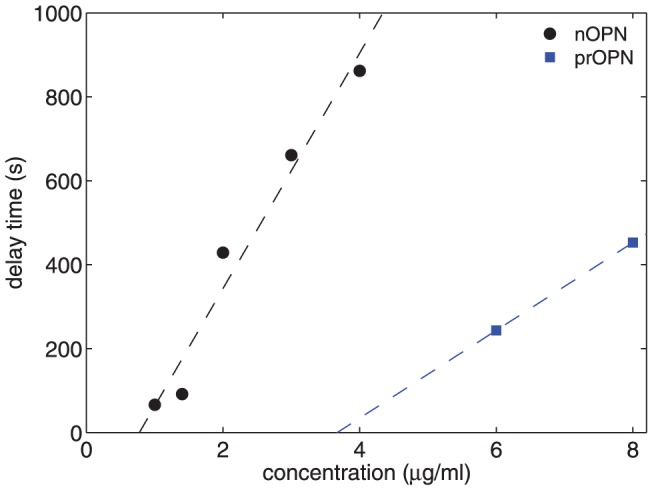
Delay time for the onset of crystal growth as a function of protein concentration. Data are plotted for both nOPN and p-rOPN. Concentrations lower than those plotted had no measurable delay. The dotted lines are linear fits to the data for each protein.

We quantified the delay in crystal nucleation by fitting a straight line to 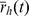 over a range of time shortly after it begins to increase significantly, and extrapolating the fit to 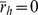. The results, shown by the solid circles in Figure 5, demonstrate that the delay time increases with the concentration of nOPN over the range studied. A straight line fit to these data suggests the existence of a threshold concentration below which the delay time will be zero; for nOPN, this threshold is approximately 0.7 µg/ml.

**Figure 6:**
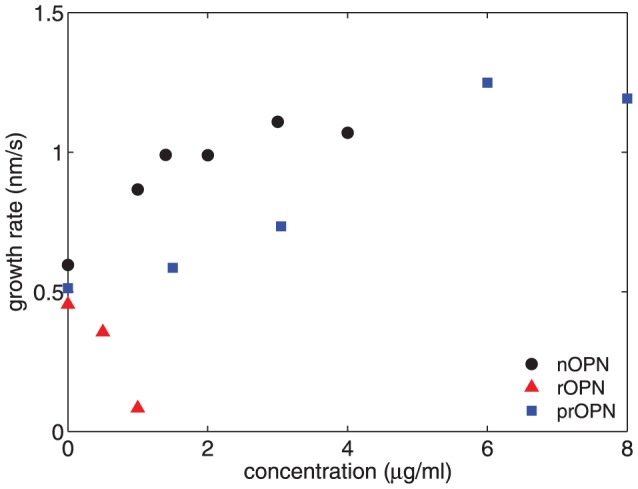
Growth rate of the precipitating partcles at 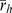 = 200 nm plotted as a function of protein concentration.

We estimated the growth rate of the precipitating crystals by fitting a straight line to the 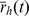 data at the time when the hydrodynamic radius of the crystals was 200 nm. The results for nOPN are plotted as circles in Figure 6. The growth rate at this crystal size increases with the concentration of nOPN, and is about a factor of two higher for [nOPN]  =  4 µg/ml than for the control experiment.

#### C: rOPN

**Figure 7:**
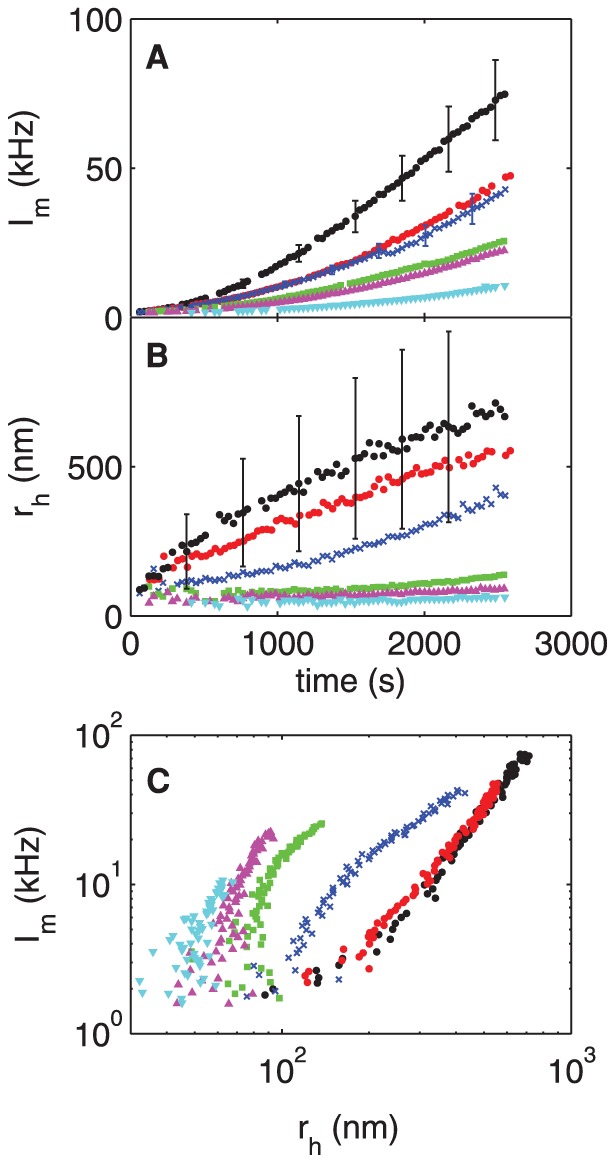
Precipitation of calcium phosphate in the presence of rOPN. (A) Mean scattered intensity and (B) hydrodynamic radius 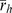 plotted as functions of time for a range of concentrations of recombinant osteopontin, rOPN. (C) shows 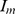 as a function of 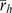. black circles: control, [rOPN]  =  0 µg/ml; red circles: 0.5 µg/ml (0.01 µM); crosses: 1 µg/ml (0.03 µM); squares: 2 µg/ml (0.06 µM); upward-pointing triangles: 3 µg/ml (0.08 µM); downward-pointing triangles: 6 µg/ml (0.17 µM). Error bars are analogous to those in Figure 4.

The scattered intensity and hydrodynamic radius measured in experiments with the recombinant OPN, which lacks the phosphorylations of the native protein, are shown in Fig. 7. As in the case of nOPN, rOPN causes a significant decrease in the intensity of scattered light relative to the control, but the behavior of the hydrodynamic radius is very different. There is no noticeable delay in the crystal growth in the presence of rOPN, but the crystals are substantially smaller and grow more slowly than in the control run at all times studied. The growth rate at a crystal size of 200 nm decreases with increasing [rOPN], as shown by the triangles in Figure 6, in contrast to the increase in growth rate observed with the native protein. For [rOPN] > 1 µg/ml (0.03 µM) the growth rate is very low and the crystals remain smaller than 100 nm for the duration of our measurements. The absence of a delay in crystal growth coupled with the decreased growth rates indicates that rOPN strongly inhibits the growth, but not the nucleation, of the calcium phosphate precipitate. The precipitate formed in the presence of rOPN was identified as HA by X-ray diffraction (results not shown).

Figure 7C shows the intensity as a function of hydrodynamic radius. In striking contrast to what was observed with nOPN, the scattered intensity at a given hydrodynamic radius is significantly higher in the presence of rOPN than in the control experiment, and increases with the concentration of the protein. This indicates that the concentration of precipitating particles is larger at a particular size than in the control, and that the concentration of particles increases with [rOPN].

#### D: p-rOPN

**Figure 8:**
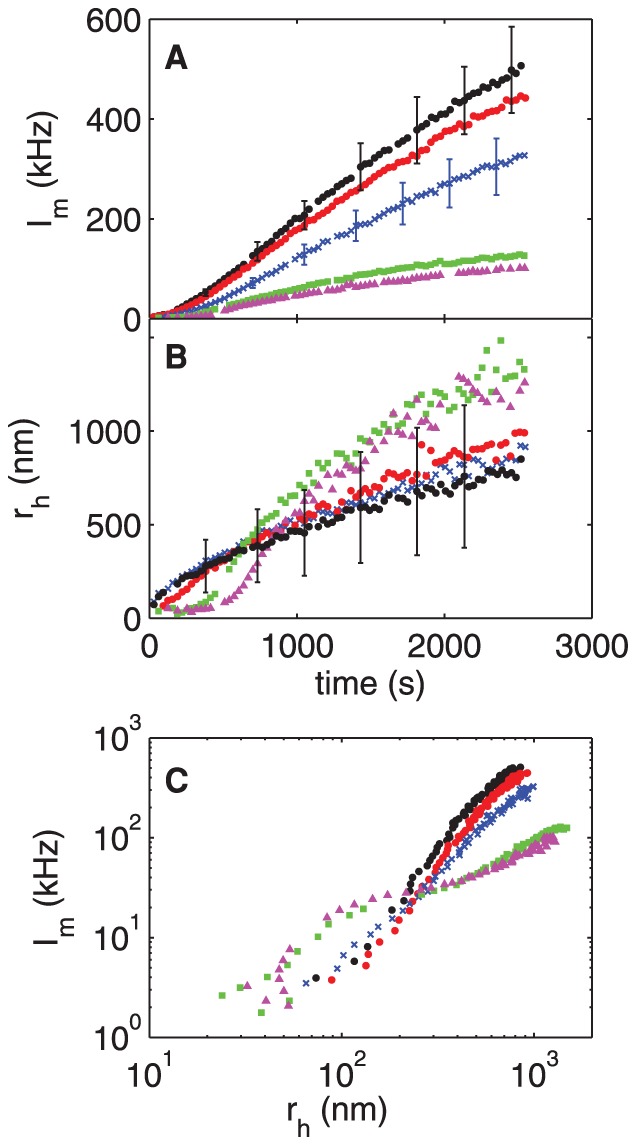
Precipitation of calcium phosphate in the presence of p-rOPN. (A) Mean scattered intensity and (B) hydrodynamic radius 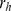 plotted as functions of time for a range of concentrations of phosphorylated recombinant osteopontin, p-rOPN. (C) shows 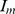 as a function of 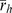. black circles: control, [p-rOPN]  =  0 µg/ml; red circles: 1.5 µg/ml; crosses: 3.1 µg/ml; squares: 6 µg/ml; upward-pointing triangles: 8 µg/ml. Error bars are analogous to those in Figure 4.

To investigate the role of phosphate groups in crystal growth kinetics, rOPN was treated with protein kinase CK2, which resulted in the addition of an average of 7–8 phosphate groups per molecule. The effects of this phosphorylated protein on the precipitation of particles from our calcium phosphate solutions are qualitatively similar to those of nOPN, as shown in Figure 8. At concentrations of 1.5 and 3.1 µg/ml, the scattered intensity is slightly smaller while the hydrodynamic radius of the crystals is similar to or slightly larger than in the control experiment, indicating a slight decrease in the concentration of the crystals. This is confirmed by the plot of 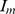 against 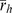 in Figure 8C. At protein concentrations of 6 and 8 µg/ml, however, a significant delay in the growth of the crystals becomes apparent, as can be seen in Figure 8B, and the scattered intensities decrease significantly. Figure 8C shows that at these higher protein concentrations, the concentration of precipitating particles increased over the control values during the delay period – i.e., when the particles are small – but is substantially lower than the control when the particles are large. The delay time is plotted as a function of protein concentration in Figure 5, and a line through the data suggests a threshold concentration for the onset of a delay in crystal growth of 3.7 µg/ml. The delay times are smaller and appear at higher concentrations for p-rOPN than for the native osteopontin. The growth rate of the crystals at 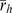 = 200 nm is plotted as a function of concentration in Figure 6. As was the case for the nOPN, the growth rate of the crystals following the delay is higher than in the control experiments.

#### E: Seeded Crystal Growth

**Figure 9:**
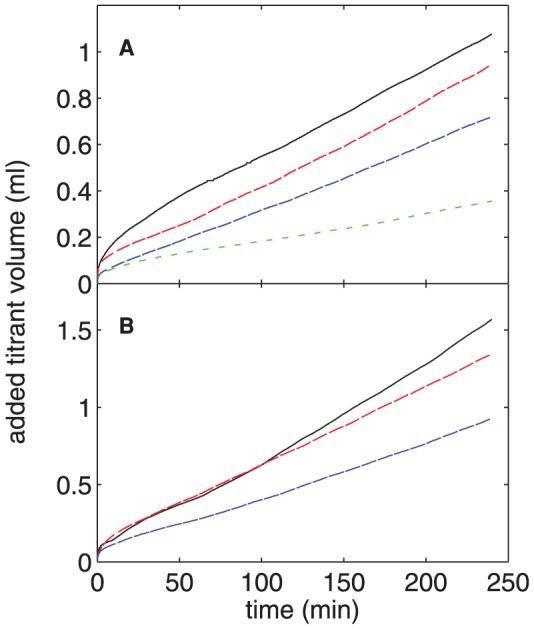
Constant-composition measurements of seeded HA crystal growth. The total volume of titrant added as a function of time in the constant-composition seeded growth experiments in the presence of (A) nOPN and (B) rOPN. The different curves correspond to different concentrations of protein. In (A), the [nOPN] is, from top to bottom, 0, 2.7, 8.0, and 15.9 µg/ml (0, 0.07, 0.21, and 0.42 µM). In (B), [rOPN] is, from top to bottom, 0, 5.0, and 15.0 µg/ml (0, 0.14, and 0.41 µM).

The constant-composition seeded-growth assay is a well-characterized protocol for assessing the effects of proteins on mineral formation [15], [21]. It is therefore useful to compare data from that experiment with our dynamic light scattering results. Figure 9A shows the total volume of titrant added to the reaction vessel as a function of time for concentrations of nOPN ranging from 0 to 16 µg/ml (0–4.2 µM). The presence of the protein results in a decrease in crystal growth that becomes more pronounced as [nOPN] is increased, as seen by the decrease in the volume of NaOH required to maintain a constant *p*H in the reaction vessel. Similar titration curves for experiments with rOPN are plotted in Figure 9B and show qualitatively similar results, that is, an increase in [rOPN] results in a decrease in crystal growth. Since the decrease in titrated volume for a given protein concentration is larger for nOPN than rOPN, nOPN is a more potent inhibitor of HA crystal growth as measured in this experiment.

**Figure 10:**
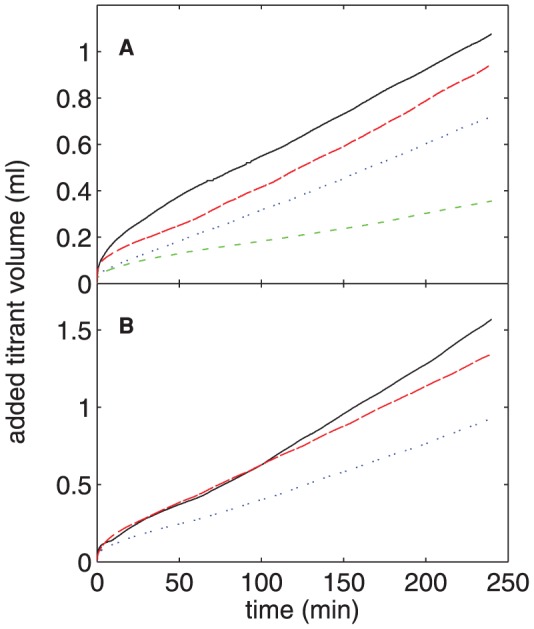
HA growth rate from the constant-composition experiments. The growth rate of the HA seed crystals relative to that in the absence of protein is plotted as a function of protein concentration for the constant-composition experiments. The dashed lines are straight lines fit to the data.

The slope of the linear portion of the titration curves, i.e., from approximately 60 min after the addition of the seed crystals until the end of the incubation period, was used to calculate the HA growth rate for each experiment. These growth rates, expressed as a percentage of that measured in the absence of protein, are plotted as a function of concentration for both nOPN and rOPN in Figure 10. Over the range of concentrations studied, the data in both cases are well described by a linear decrease in relative growth rate with increasing concentration; fits are plotted in Figure 10. The protein concentration that resulted in 50% inhibition of growth rate (referred to as IC_50_) was determined from these fits to be 11.9 µg/ml (0.317 µM) for nOPN and 22.7 µg/ml (0.628 µM) for rOPN.

## Discussion

Based on the DLS experiments, all the variants of OPN that were studied have an inhibitory effect on the precipitation of calcium phosphate particles from a supersaturated solution. The effects were different for the three isoforms, however. The native OPN caused a delay in the nucleation of the particles and a substantial reduction in particle concentration relative to the control. The smaller number of particles that did form, however, grew more quickly than in the control and reached sizes approaching 1 µm by the end of the experiment. The lag time beyond which rapidly growing particles appear increased linearly with the concentration of nOPN, once it exceeded a threshold value. The observed decrease in particle concentration indicates that nOPN strongly inhibits the formation of the particles, either by preventing their nucleation in the first place or by preventing small nucleated clusters from growing large enough to be detected in our experiments.

In contrast, rOPN significantly slowed the growth of the precipitate particles and at concentrations exceeding 1 µg/ml the particle size remained less than 100 nm over the course of the 40-minute incubation. rOPN did not induce a measureable lag time, suggesting that it has no effect on nucleation. Since rOPN lacks all post-translational modifications, these results suggest that the “core” protein has the ability to inhibit growth.

As outlined in the Introduction, post-translational modifications contribute to the ability of OPN to inhibit HA formation. While other modifications may also play a role, a number of studies have shown that the phosphates attached to nOPN substantially enhance the inhibitory activity of the protein [8], [9] or related peptides [14], [15]. A recent study using DLS to study the effects of a 14-residue osteopontin-derived peptide on calcium phosphate precipitation showed similar roles of phosphate in modulating mineral formation [39]. The effect of the phosphate groups is also evident from our DLS results. The behavior of the phosphorylated recombinant OPN (p-rOPN) is qualitatively very similar to that of the native protein: it causes a concentration-dependent delay in particle formation and a decrease in the concentration of large particles. Both nOPN and p-rOPN behave quite differently from the non-phosphorylated rOPN. p-rOPN is not as potent an inhibitor as nOPN, however, as evidenced by the facts that the lag times induced by p-rOPN are shorter than for nOPN at a given protein concentration and the threshold concentration at which the delay first appears is higher for p-rOPN. There are several possible reasons for the stronger inhibitory activity of nOPN. For one thing, nOPN has more phosphates. nOPN also has additional modifications, including attached sugars, some of which are sialic acids. In addition, the locations of the phosphorylations along the protein may be different in nOPN and p-rOPN. Whatever the reason, our data clearly indicate that attached phosphates on OPN contribute significantly to the inhibition of HA nucleation.

The role of phosphate groups in the interaction between OPN and biominerals has been studied using molecular dynamics simulations. In general, these simulations suggest that the adsorption of peptides based on sequences in rat bone OPN to the Ca-rich {100} faces of both HA [15] and calcium oxalate monohydrate [40] is driven by electrostatics and is largely independent of amino-acid sequence. Therefore, phosphate groups increase the electronegativity of OPN, resulting in a stronger interaction with cationic crystal faces.

There are several possible explanations for the inhibition of nucleation displayed by both nOPN and, at higher concentrations, p-rOPN. One possibility is that the negatively charged OPN may bind large amounts of positively charged calcium ions, effectively lowering the ion concentration in the solution and reducing the driving force for precipitation. OPN is known to bind calcium, with measured values ranging from 8 to 50 Ca^2+^ ions per molecule, depending on the conditions used in the assay and the source of OPN [41], [42]. Even at the highest measured level, however, the binding of Ca^2+^ to OPN in the incubation mixture would lower the effective calcium concentration by only 1.5 µM. This small decrease is unlikely to have any measureable effect on precipitation kinetics.

Alternatively, OPN may bind to small (i.e., smaller than the critical size for nucleation) calcium phosphate clusters, preventing them from growing large enough to become stable and precipitate out of solution and resulting in their eventual re-dissolution. It is also possible that these calcium phosphate clusters may in fact be stabilized by OPN, but prevented from growing further. Since some OPN remains bound to these clusters, the concentration of free OPN is reduced, allowing some clusters to eventually grow and transform into crystalline HA. It is generally regarded that there is a finite probability of critical nucleus formation at any given concentration of Ca^2+^ and phosphate. In the light scattering experiments, the concentration of ions in solution will decrease as they are incorporated into the growing particles and the rate of formation of new clusters will thus decrease over time. If nucleation is blocked, however, as is the case for the phosphorylated isoforms of OPN but not for rOPN, the concentration of free Ca^2+^ and phosphate will remain essentially constant. This implies that the few nuclei that escape the inhibitory effects of OPN will, over time, grow faster than in the absence of the protein because the ion concentrations are higher than they would be if protein was present. Consistent with our experiments, the end result would be a lower number of particles that grow faster than in the absence of the inhibitory protein.

The constant-composition experiments showed that both nOPN and rOPN inhibited the growth of seeded HA crystals, with nOPN being the more potent inhibitor as measured by this well-established assay. The constant-composition assay and the DLS experiments are performed under very different conditions: in the former case, the composition of the solution is kept fixed as pre-existing seed crystals grow, while in the latter, the composition changes as new crystals nucleate and grow. As a result, the two experiments cannot be directly compared. Nonetheless we can state that the constant-composition data are broadly consistent with the results of our light scattering experiments, in that both isoforms of the protein reduce the amount of calcium and phosphate removed from the solutions. Very little additional information can be derived from the constant-composition data. In contrast, the light scattering data make it clear that nOPN (and p-rOPN at higher concentrations) act to inhibit the nucleation of calcium phosphate particles, while rOPN inhibits growth but has no significant effect on nucleation. The DLS measurements thus provide important information that is not available from other, more established techniques for studying the effects of proteins on crystal formation. Our results demonstrate that it is relatively straightforward to use dynamic light scattering to measure the characteristic size of growing mineral particles in real time. The effects of modulatory proteins on growth kinetics and, at least semi-quantitatively, particle concentrations, can be observed directly, making it possible to distinguish between different types of inhibitory behavior.

## Conclusions

We have used dynamic light scattering to study the effects of OPN and related proteins on the precipitation of calcium phosphate particles from solution. Analysis of the light scattering data gives the characteristic size of the precipitating particles as a function of time, and provides some information about their concentration. This allows us to study the effects of the proteins on both the nucleation of calcium phosphate particles and their subsequent growth. We found that nOPN strongly inhibits the nucleation of calcium phosphate particles and induces a delay in the appearance of precipitate which increases with increasing protein concentration. The few particles which do nucleate then grow faster than those that form in the absence of protein. In contrast, rOPN, which lacks the posttranslational modifications of the native protein, appears to enhance nucleation but strongly inhibits the subsequent growth of the particles. p-rOPN, has an effect similar to but weaker than nOPN. These results indicate that phosphorylations play a major role in the inhibition of nucleation by OPN, and that the growth of the calcium phosphate particles is modulated by the acidic OPN polypeptide even in the absence of posttranslational modifications. Our results are consistent with those obtained from a constant-concentration assay, but the light scattering experiments provide much more information about the growth kinetics and allow us to distinguish the different modes of inhibition displayed by the different isoforms of the protein.

## References

[1] Mann S (2001) Biomineralization: Principles and Concepts in Bioinorganic Materials Chemistry. Oxford: Oxford University Press.

[2] George A, Veis A (2008) Phosphorylated Proteins and Control over Apatite Nucleation, Crystal Growth, and Inhibition. Chem. Rev. 108: 4670–4693. 10.1021/cr0782729 PMC2748976 18831570

[3] Addadi L, Weiner S, Geva M (2001) On how proteins interact with crystals and their effect on crystal formation. Zeitschrift fur Kardiologie 90, Suppl. 3 92–98. 10.1007/s003920170049 11374040

[4] De Yoreo JJ, Dove PM (2004) Shaping crystals with biomolecules. Science 306: 1301–1302. 15550649 10.1126/science.1100889

[5] Giachelli CM (2005) Inducers and inhibitors of biomineralization: lessons from pathological calcification. Orthod. Craniofac. Res 8: 229–231. 16238602 10.1111/j.1601-6343.2005.00345.x

[6] Lowenstam HA, Weiner S (1989) On Biomineralization, Oxford: Oxford University Press.

[7] Shiraga H, Min W, Van Dusen WJ, Clayman MD, Miner D, et al (1992) Inhibition of calcium oxalate crystal growth in vitro by uropontin: Another member of the aspartic acid-rich protein superfamily. Proc. Nat. Acad. Sci. USA 89: 426–430. 10.1073/pnas.89.1.426 PMC48250 1729712

[8] Boskey AL, Maresca M, Ulirich W, Doty SB, Butler WT, et al (1993) Osteopontin-hydroxyapatite interactions in vitro. Inhibition of hydroxyapatite formation and growth in a gelatin-gel. Bone Miner 22: 147–159. 8251766 10.1016/s0169-6009(08)80225-5

[9] Hunter GK, Kyle CL, Goldberg HA (1994) Modulation of Crystal Formation by Bone Phosphoproteins: Structural Specificity of the Osteopontin-Mediated Inhibition of Hydroxyapatite Formation. Biochem. J 300: 723–728. 8010953 10.1042/bj3000723 PMC1138226

[10] Fisher LW, Torchia DA, Fohr B, Young MF, Fedarko NS (2001) Flexible structures of SIBLING proteins, bone sialoprotein and osteopontin. Biochem. Biophys. Res. Comm 280: 460–465. 11162539 10.1006/bbrc.2000.4146

[11] Butler WT (1995) Structural and functional domains of osteopontin. Ann. N.Y. Acad. Sci 760: 6–11. 7785926 10.1111/j.1749-6632.1995.tb44615.x

[12] Keykhosravani M, Doherty-Kirby A, Zhang C, Brewer D, Goldberg HA, et al (2005) Comprehensive identification of post-translational modifications of rat bone osteopontin by mass spectrometry. Biochemistry 44: 6990–7003. 15865444 10.1021/bi050109p

[13] Grohe B, Taller A, Vincent PL, Tieu LD, Rogers KA, et al (2009) Crystallization of calcium oxalates is controlled by molecular hydrophilicity and specific polyanion-crystal interactions. Langmuir 25: 11635–11646. 19725562 10.1021/la901145d

[14] Pampena DA, Robertson KA, Litvinova O, Lajoie G, Goldberg HA, et al (2004) Inhibition of hydroxyapatite formation by osteopontin phosphopeptides. Biochem. J 378: 1083–1087. 14678013 10.1042/BJ20031150 PMC1224036

[15] Azzopardi PV, O’Young J, Lajoie G, Karttunen M, Goldberg HA, et al (2010) Roles of Electrostatics and Conformation in Protein-Crystal Interactions. PLoS One. 5: : e9330 1–11. 10.1371/journal.pone.0009330 PMC2824833 20174473

[16] Hincke MT, St. Maurice M (2000) Phosphorylation-dependent modulation of calcium carbonate precipitation by chicken eggshell matrix proteins. In: Goldberg M, Boskey A, Robinson C, editors. Chemistry and biology of mineralized tissues, Rosemount: American Academy of Orthopaedic Surgeons. pp. 13–17.

[17] Hoyer JR, Asplin JR, Otvos L (2001) Phosphorylated osteopontin peptides suppress crystallization by inhibiting the growth of calcium oxalate crystals. Kidney International 60: 77–82. 11422738 10.1046/j.1523-1755.2001.00772.x

[18] Gericke A, Qin C, Spevak L, Fujimoto Y, Butler WT, et al (2005) Importance of phosphorylation for osteopontin regulation of biomineralization. Calcif. Tissue Int 77: 45–54. 16007483 10.1007/s00223-004-1288-1 PMC1451414

[19] Wang L, Guan X, Tang R, Hoyer JR, Wierzbicki A, et al (2008) Phosphorylation of osteopontin is required for inhibition of calcium oxalate crystallization. J. Phys. Chem.B 112: 9151–9157. 18611047 10.1021/jp804282u PMC2743538

[20] Hunter GK, Hauschka PV, Poole AR, Rosenberg LC, Goldberg HA (1996) Nucleation and inhibition of hydroxyapatite formation by mineralized tissue proteins. Biochem. J 317: 59–64. 8694787 10.1042/bj3170059 PMC1217486

[21] Tomson MB, Nancollas GH (1978) Mineralization kinetics: a constant composition approach. Science 200: 1059–1060. 17740700 10.1126/science.200.4345.1059

[22] O’Young J, Liao Y, Xiao Y, Jalkanen J, Lajoie G, et al (2011) Matrix Gla protein inhibits ectopic calcification by a direct interaction with hydroxyapatite crystals. J. Am. Chem. Soc 133: 18406–18412. 21961692 10.1021/ja207628k

[23] Boskey AL (1992) Mineral-matrix interactions in bone and cartilage. Orthop. Relat. Res 281: 244–274. 1323440

[24] Onuma K, Oyane A, Tsutsui K, Tanaka K, Treboux G, et al (2000) Precipitation kinetics of hydroxyapatite revealed by the continuous-angle laser light-scattering technique. J. Phys. Chem.B 104: 10563–10568.

[25] Onuma K, Ito A (1998) Cluster growth model for hydroxyapatite. Chem. Mater 10: 3346–3351.

[26] Oyane A, Onuma K, Kokubo T, Ito A (1999) Clustering of calcium phosphate in the system CaCl_2_-H_3_PO_4_-KCl-H_2_O. J. Phys. Chem.B 103: 8230–8235.

[27] Heiss A, DuChesne A, Denecke B, Grötzinger J, Yamamoto K, et al (2003) Structural basis of calcium inhibition by α_2_-HS glycoprotein/fetuin A. J. Biol. Chem 278: 13333–13341. 12556469 10.1074/jbc.M210868200

[28] Kamiya N, Tsunomori F, Kagi H, Notsu K (2011) Dynamic light scattering study of the inhibiting effect on crystal growth of calcium carbonate. Bull. Chem. Soc. Jpn 84: 344–348.

[29] Zhang Q, Domenicucci C, Wrana JL, Sodek J (1990) Characterization of fetal porcine bone sialoproteins, secreted phosphoprotein I (SPPI, osteopontin), bone sialoprotein, and a 23-kDa glycoprotein. Demonstration that the 23-kDa glycoprotein is derived from the carboxyl terminus of SPPI. J. Biol. Chem 265: 7583–7589. 2332443

[30] Baht GS, O’Young J, Borovina A, Chen H, Tye CE, et al (2010) Phosphorylation of Ser^136^ is critical for potent bone sialoprotein-mediated nucleation of hydroxyapatite crystals. Biochem. J 428: 385–395. 20377527 10.1042/BJ20091864

[31] Flemming RL (2007) Micro X-ray Diffraction (μXRD): A versatile technique for characterization of Earth and planetary materials. Can. J. Earth Sci 44: 1333–1346.

[32] Berne BJ, Pecora R (1976) Dynamic Light Scattering: with Applications to Chemistry, Biology, and Physics. New York: Wiley.

[33] Xu R (2000) Particle characterization: light scattering methods. Dordrecht: Kluwer.

[34] ALV-GmbH website. Available: http://www.alvgmbh.de/About_ALV/about_alv.html. Langen, Germany.

[35] Frisken BJ (2001) Revisiting the method of cumulants for the analysis of dynamic light-scattering data. Applied Optics 40: 4087–4091. 18360445 10.1364/ao.40.004087

[36] Nancollas GH, Mohan MS (1970) The growth of hydroxyapatite crystals. Arch. Oral Biol 15: 731–745. 5272549 10.1016/0003-9969(70)90037-3

[37] Provencher SW (1982) CONTIN: A general purpose constrained regularization program for inverting noisy linear algebraic and integral equations. Comput. Phys. Comm 27: 229–242.

[38] Marino I-G (2007) Available: http://www.mathworks.com/matlabcentral/fileexchange/6523-rilt. Accessed 2010 Apr 5.

[39] Li S, Wang L (2012) Phosphorylated osteopontin peptides inhibit crystallization by resisting the aggregation of calcium phosphate nanoparticles. Cryst. Eng. Comm 14: 8037–8043.

[40] Grohe B, O’Young J, Ionescu DA, Lajoie G, Rogers KA, et al (2007) Control of calcium oxalate crystal growth by face-specific adsorption of an osteopontin phosphopeptide. J. Amer. Chem. Soc 129: 14946–14951. 17994739 10.1021/ja0745613

[41] Singh K, Deonarine D, Shanmugam V, Senger DR, Mukherjee AB, et al (1993) Calcium-binding properties of osteopontin derived from non-osteogenic sources. J. Biochem 114: 702–707. 8113224 10.1093/oxfordjournals.jbchem.a124240

[42] Chen Y, Bal BS, Gorski JP (1992) Calcium and collagen binding properties of osteopontin, bone sialoprotein, and bone acidic glycoprotein-75 from bone. J. Biol. Chem 267: 24871–24878. 1447223

